# Axillary Artery Injury with Intact Radial Pulse following Fracture-Dislocation of Shoulder: A Case Report

**DOI:** 10.5704/MOJ.1911.011

**Published:** 2019-11

**Authors:** A Rajeev, G Timmons

**Affiliations:** Department of Orthopaedics, Gateshead Health NHS Foundation Trust, Gateshead, United Kingdom; * Department of Radiology, Gateshead Health NHS Foundation Trust, Gateshead, United Kingdom

**Keywords:** axillary artery injury, intact radial pulse, shoulder dislocation

## Abstract

The occurrence of axillary artery injury following proximal humerus fracture dislocation in elderly patient with low velocity fall is uncommon. The patient could have diverse clinical presentations in spite of intact peripheral pulses. We report the case of an 85-year-old lady who presented to our emergency department with greater tuberosity fracture of the humerus with dislocation of the right shoulder. After closed manipulative reduction of the dislocation, it was observed that the patient had brachial plexus palsy with intact radial pulse. An expanding swelling and bruise around the shoulder was noted and a steady drop in haemoglobin level. CT angiogram revealed avulsion of the posterior circumflex artery which was then treated successfully with stenting.

## Introduction

The fractures of the proximal humerus accounts for 5% of all long bone fractures^1^. The association of axillary artery injury is not common with these injuries especially in elderly patients. The reasons for low occurrence of vascular injuries with fractures of proximal humerus and shoulder dislocation is due to the fact that there are no compact compartments in axilla and the tissues are very lax and accommodative. The early diagnosis of the potentially serious injury is important because of the high incidence of limb ischemia, soft tissue contracture, amputation and death.

We report a rare case of fracture-dislocation of shoulder with axillary artery injury but with intact radial pulse which led to a delay in the diagnosis of axillary artery injury.

## Case Report

An 85-year-old right-hand dominant lady was admitted to the emergency care department after a fall. She had pain, deformity and restriction of movements of her right shoulder joint. She also complained of right chest pain. There was no significant past medical history and she was not on any oral anticoagulants.

**Fig. 1:**
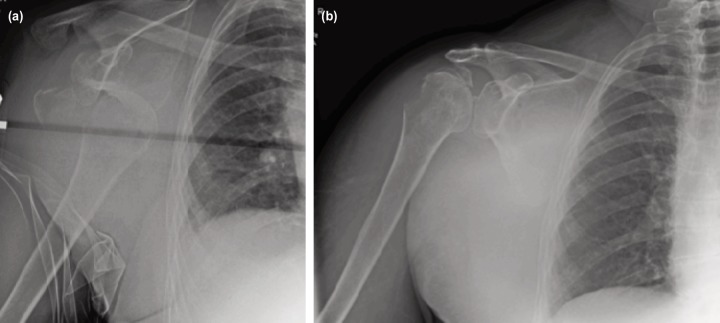
Radiographs of right shoulder (a) AP view showing anterior fracture dislocation, (b) Post-manipulation showing reduction of dislocation and displaced greater tuberosity fracture fragments.

On examination, there was deformity consistent with dislocation of the right shoulder. Movements of the right shoulder were restricted due to pain. The radial pulse was present and there was no distal neurovascular deficit. Radiograph revealed anterior dislocation of the right shoulder with comminuted fracture of the greater tuberosity (Fig. 1a). Chest radiograph revealed fractures of 2nd, 3rd and 4th ribs on the right side. The haemoglobin level was 12gm/dl.

After resuscitation, the patient`s right shoulder was reduced with closed manipulation under sedation and Entonox inhalation, confirmed with check post-reduction radiograph, with greater tuberosity fragment mildly displaced (Fig. 1b). Examination of the right upper extremity after reduction revealed intact radial artery pulse and evidence of posterior cord palsy of the brachial plexus. The patient was transferred to high dependency unit (HDU) for monitoring and observation.

**Fig. 2:**
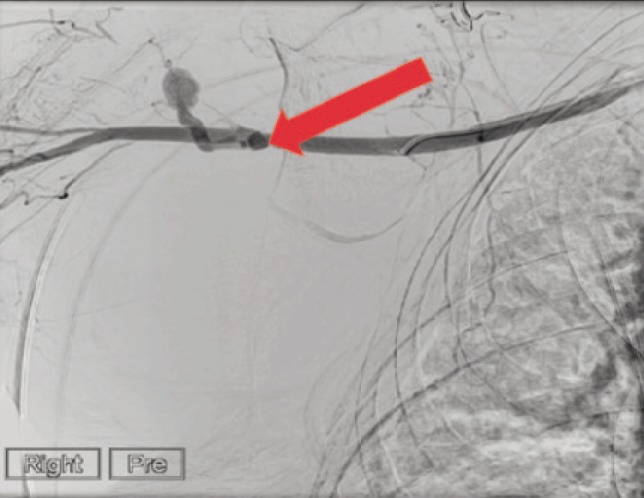
CT Angiogram showing avulsion of posterior circumflex

After six hours of admission to HDU, the patient’s haemoglobin level continued to drop steadily, and she was transfused with six units of blood over a period of ten hours. The radial artery pulse was present throughout this time. However, there was continuous drop of blood pressure and haemoglobin level despite aggressive resuscitation. CT angiogram showed a disruption of the intima at a single point of the right axillary artery and a large pseudo aneurysm due to avulsion of posterior circumflex artery with a large hematoma (Fig. 2).

**Fig. 3:**
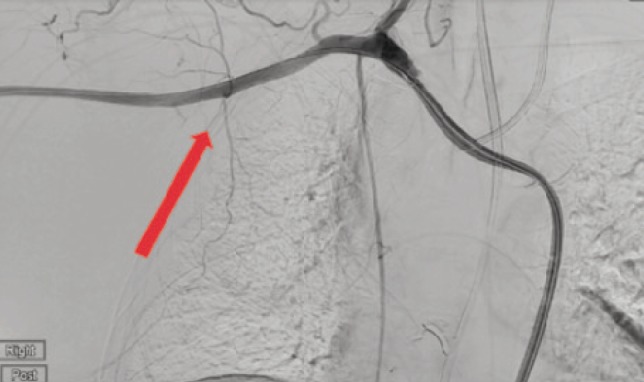
Post-stenting angiogram showing no leak. branch from axillary artery.

The Interventional Radiology department intervened and following selective cannulation of the right subclavian artery, a 6cm Polytetrafluorethylene- (PTFE) covered stent (Atrium Advanta V12) was deployed across the traumatic pseudo-aneurysm of the right axillary artery with good completion of the angiography and with no further evidence of leak (Fig. 3).

The patient then developed pulmonary atelectasis and consolidation of the right lung. She was treated with intravenous Tazocin. Over the next two weeks she continued to improve but on the 14th day died due to myocardial infarction.

## Discussion

Even though the incidence of axillary artery injury is rare with fractures and dislocation of proximal humerus, the factors that predispose to arterial injury are elderly age, osteoporosis, wide displacement of fracture fragments and atherosclerosis. Our patient had all the predisposing factors for the axillary artery injury.

The clinical presentation may be acute or delayed depending on the vascular injury mechanism. The various mechanisms postulated are direct arterial injury due to sharp fracture fragments, avulsion of arterial branches or rupture and intimal tears. The distal peripheral pulses including the radial artery may be palpable initially. Intimal tears can lead to delayed presentation due to secondary thrombosis, with palpable peripheral pulses on initial presentation. Effective collateral circulation at shoulder level can result in the presence of peripheral radial artery pulse despite axillary artery damage^2^. The other causes for an intact radial pulse in axillary artery injury are intimal tears with intra-arterial hematoma formation and thrombosis. Our patient had avulsion of posterior circumflex artery and intimal tears of axillary artery.

Kelley *et al* described a diagnostic triad for the detection of axillary artery injuries,which included proximal humerus fractures or dislocations, expanding hematoma and reduction in the volume of radial pulse^3^. Our patient had two of these three diagnostic triads, namely shoulder trauma and expanding hematoma.

Triple phase CT Angiography is the examination of choice but doppler ultrasound is also a useful tool^4^. Pezeshki *et al* in their study comparing color doppler ultrasound and angiography showed that the doppler had a sensitivity of 95% and specificity of 98% in the diagnosis of arterial injury. Positive and negative predictive values of doppler ultrasound were 92.5% and 94.2%, respectively^4^. Deploying a covered stent in this scenario is relatively straightforward and avoids complex surgery as the surgical access to this area is restricted. The treatment of these potentially serious injuries is open repair with vein grafts if open reduction and internal fixation or arthroplasty are contemplated. But if the fracture or dislocation is treated non-operatively then angioplasty or stenting can be carried out. In our patient since we had decided to treat the fracture-dislocation non-operatively she underwent stenting procedure, with good results and no further arterial leak.

In patients with proximal humerus fractures the incidence of brachial plexus injury is about 6%^5^ but in the presence of axillary artery injury it goes up to 50%^1^. McLaughlin *et al* found that arterial injury occurred in 84% patients older than 50 years, 53% were associated with brachial plexus injury and 21% resulted in upper extremity amputation^1^. In our patient there were signs and symptoms of involvement of both the brachial plexus and axillary artery injury after the closed manipulation of the fracture dislocation. The probable causes for neurovascular injury in our patient could be from the manipulation causing of avulsion of nerve roots, neuropraxia, and pressure effects of hematoma from arterial bleed.

In elderly patients with fracture dislocations of shoulder, the presence of brachial plexus and vascular injuries should strengthen the need for a detailed neurovascular examination. In such cases axillary artery injury should be suspected in spite of an intact radial pulse especially in cases of expanding swelling around the shoulder or anterior chest wall, steady drop in hemoglobin, fluctuating recordings of blood pressure. There should be a high index of suspicion for branch avulsions and intimal tears of the axillary artery. Prompt and early vascular surgery consultation is advised in these circumstances. Closed manipulative reduction of fracture-dislocation of shoulder in the emergency room is then not recommended and it should be performed with gentle manipulation under general anesthesia in the operation theatre.
